# Obesity and Risk of Recurrence in Patients With Breast Cancer Treated With Aromatase Inhibitors

**DOI:** 10.1001/jamanetworkopen.2023.37780

**Published:** 2023-10-13

**Authors:** Sixten Harborg, Deirdre Cronin-Fenton, Maj-Britt Raaby Jensen, Thomas P. Ahern, Marianne Ewertz, Signe Borgquist

**Affiliations:** 1 Department of Oncology, Aarhus University Hospital/Aarhus University, Aarhus, Denmark; 2 Department of Clinical Epidemiology, Aarhus University, Aarhus N, Denmark; 3 Danish Breast Cancer Group, Copenhagen University Hospital, Copenhagen, Denmark; 4 Department of Surgery, Larner College of Medicine, University of Vermont, Burlington, Vermont; 5 Oncology Research Unit, Department of Clinical Research, University of Southern Denmark, Odense C, Denmark; 6 Department of Clinical Sciences, Lund, Oncology, Lund University, Sweden

## Abstract

**Importance:**

Clinical studies confirm that obesity is a risk factor for recurrence in postmenopausal women with hormone receptor–positive (HR+) breast cancer. Evidence suggests that women with obesity do not obtain similar protection from aromatase inhibitors as women with healthy weight.

**Objective:**

To examine the associations of body mass index (BMI) with recurrence.

**Design, Setting, and Participants:**

The cohort study was conducted using data from the Danish Breast Cancer Group and enrolled postmenopausal women diagnosed with stage I to III HR+ breast cancer from 1998 through 2016. Data analysis was conducted from November 2022 to April 2023.

**Exposures:**

BMI was classified as (1) healthy weight (18.5-24.9), (2) overweight (25.0-29.9), (3) obesity (30.0-34.9), and (4) severe obesity (≥35.0) using the World Health Organization guidelines. Healthy weight was considered the reference group in statistical analyses.

**Main Outcomes and Measures:**

Follow-up began 6 months after breast cancer surgery and continued until the first event of recurrence, contralateral breast cancer, new primary malignant neoplasm, death, emigration, end of clinical follow-up at 10 years, or September 25, 2018. Cox regression was used to estimate crude and adjusted hazard ratios with 95% CIs, adjusting for patient, tumor, and treatment characteristics.

**Results:**

A total of 13 230 patients (median [IQR] age at diagnosis, 64.4 [58.6-70.2] years) with information on BMI were enrolled. There were 1587 recurrences with a median (IQR) potential estimated follow-up of 6.2 (3.6-8.5) years. Multivariable analyses revealed increased recurrence hazards associated with obesity (adjusted hazard ratio, 1.18 [95% CI, 1.01-1.37]) and severe obesity (adjusted hazard ratio, 1.32 [95% CI, 1.08-1.62]) vs patients with healthy weight. Patients with overweight had a greater risk, but the results were not statistically significant (adjusted hazard ratio, 1.10 [95% CI, 0.97-1.24]).

**Conclusions and Relevance:**

In this study, obesity was associated with an increased risk of breast cancer recurrence among postmenopausal patients with HR+ early-stage breast cancer treated with aromatase inhibitors. Physicians should be aware of the significance of obesity on breast cancer outcomes to secure optimal treatment benefit in all patients.

## Key Points

**Question:**

Is obesity associated with increased recurrence risk in patients with breast cancer treated with adjuvant aromatase inhibitors?

**Findings:**

In this nationwide cohort study of 13 230 patients with hormone receptor–positive breast cancer, 1587 had a recurrence over 6.2 years of follow-up. The risk of recurrence was higher among patients with obesity and severe obesity compared with those with healthy weight.

**Meaning:**

This study suggests that obesity is associated with an increased risk of recurrence among patients with hormone receptor–positive breast cancer treated with aromatase inhibitors, highlighting the need for optimization of care in patients with breast cancer and obesity.

## Teaser

This cohort study examines the associations of body mass index (BMI) with recurrence among patients with breast cancer treated with aromatase inhibitors in Denmark.

## Introduction

Body mass index (BMI; calculated as weight in kilograms divided by height in meters squared) is the standard metric for classifying adiposity. High BMI has repeatedly been shown to be associated with a poorer prognosis of early breast cancer.^1,2,3^ Yet, the mechanisms by which obesity affects prognosis remain unclear. One mechanism may be reduced clinical efficacy of adjuvant treatment (eg, endocrine treatment with aromatase inhibitors [AI] for hormone receptor–positive [HR+] postmenopausal breast cancer) in patients with obesity.^4^ HR+ breast cancer is the most common subtype of breast cancer among postmenopausal women, accounting for 80% of all cases.^5^ The adjuvant endocrine therapy of choice for this patient group is an AI.^6^ Aromatase is highly expressed in adipose tissue, where it catalyzes the biotransformation of androgens into estrogen; it is also the target enzyme for AIs in the context of treating HR+ breast cancer in postmenopausal women.^7,8^

Previous investigations suggest that AIs are less effective in suppressing estradiol production in patients with obesity than in women with healthy weight.^9,10^ This hypothesis was investigated in 2 phase 3 endocrine therapy trials testing AI vs tamoxifen treatment in the adjuvant setting.^4,11^ The analysis nested in the Arimidex, Tamoxifen, Alone or in Combination (ATAC) trial showed that patients with obesity receiving AIs had more recurrences than patients with healthy weight receiving AIs, suggesting a relative clinical efficacy of AI treatment depending on body weight.^4^ This finding was not evident among the patients receiving tamoxifen in the ATAC trial, leaving the question as to whether treatment with tamoxifen might be more suitable for patients with obesity. In the similar adjuvant BIG 1-98 trial, no attenuated AI response among patients with obesity was observed, and treatment with AIs seemed superior to tamoxifen treatment independently of weight status.^11^ Thus, more evidence is needed about the efficacy of AI-mediated estrogen suppression in patients with breast cancer and obesity.

High BMI has been identified as a risk factor for developing postmenopausal breast cancer, and at the same time, obesity can influence HR status, with a higher likelihood of HR+ tumors in people with obesity.^12^ As a consequence of the ongoing obesity epidemic,^13^ more people with obesity will be treated with AIs. People with obesity also tend to have larger tumors and increased lymph node involvement, factors associated with a poorer survival.^14,15^ In 2022, the evidence for an association between body fatness and breast cancer recurrence was judged limited by the Global Cancer Update Programme, calling for more studies on this matter.^16^ Furthermore, obesity can affect the response to cancer treatment, as obesity alters drug metabolism and distribution, potentially affecting the efficacy of, for example, endocrine therapy.^17^ Given obesity’s ability to alter drug metabolism and its impact on breast cancer survival rates, studying the association of BMI with breast cancer recurrence can contribute to the development of more personalized treatment strategies.^18^ Enhanced understanding of how a patient’s BMI interacts with their breast cancer can help health care professionals tailor treatment plans to achieve better outcomes.

We hypothesized that adjuvant treatment with AIs for postmenopausal patients with early-stage HR+ breast cancer and obesity is not as beneficial in terms of clinical outcomes as for patients with a healthy weight. We studied the association between BMI and breast cancer recurrence in a national Danish cohort.

## Methods

We conducted a nationwide, population-based cohort study using Danish clinical and administrative registries. This study was approved by the Danish Breast Cancer Group and the Danish Data Protection Agency and adheres to the General Data Protection Regulations.^19^ The study is based on routinely collected registry data and therefore, according to Danish regulations, does not require separate ethical approval or informed consent. The study is reported in accordance with the Strengthening the Reporting of Observational Studies in Epidemiology (STROBE) guidelines.^20^

### Data Sources

In this study, all data sources were linked at the individual level using the civil personal registration number (CPR No.). The CPR No. is a unique identifier assigned to all Danish residents upon birth or immigration.

#### The Danish Breast Cancer Group Clinical Database

The clinical database of the Danish Breast Cancer Group (DBCG)^21^ covers the entire Danish female population and includes data on invasive breast cancers diagnosed in Denmark since 1977 with a completeness of more than 95%.^22^ All hospital departments of the Danish health care system involved in the diagnosis, treatment, and follow-up of breast cancer submit patient data to DBCG using standardized forms.^23^ The information retrieved from the DBCG registry for this study included patient age at diagnosis, BMI, histologic tumor type and grade, lymph node status, tumor size, estrogen receptor status, human epidermal growth factor receptor 2 (*ERBB2* [formerly *HER2*/*neu*]) status, type of primary surgery, adjuvant therapy (chemotherapy, endocrine therapy, and radiotherapy), and time to recurrence.

#### The Danish Civil Registration System

Since 1968, the Danish Civil Registration System has collected migration and vital status data on the Danish population.^24^ As with all Danish registries, the Civil Registration System includes the CPR No., allowing linkage of independent registries at the individual level with 100% accuracy.

#### The Danish National Patient Registry

The Danish National Patient Registry, established in 1977, includes information on hospital admissions, discharges, and since 1995, emergency department visits and outpatient visits.^25^ For each hospital encounter, 1 action diagnosis and up to 20 other diagnoses are recorded.^26^ Data on comorbid diseases present at the time of breast cancer surgery were gathered from the Danish National Patient Registry through linkage via the CPR No. and summarized using the Charlson Comorbidity Index (CCI),^27^ excluding breast cancer and nonmelanoma skin cancer from the list of contributing conditions.

#### The Danish Anesthesia Database

The Danish Anesthesia Database (established 2004) includes data on individuals undergoing anesthesia in Denmark, covering approximately 70% of all anesthesiology procedures.^28^ Data on height and weight were obtained from the Danish Anesthesia Database to supplement missing BMI data ascertained from the DBCG database.

### Study Population

**Figure 1.**
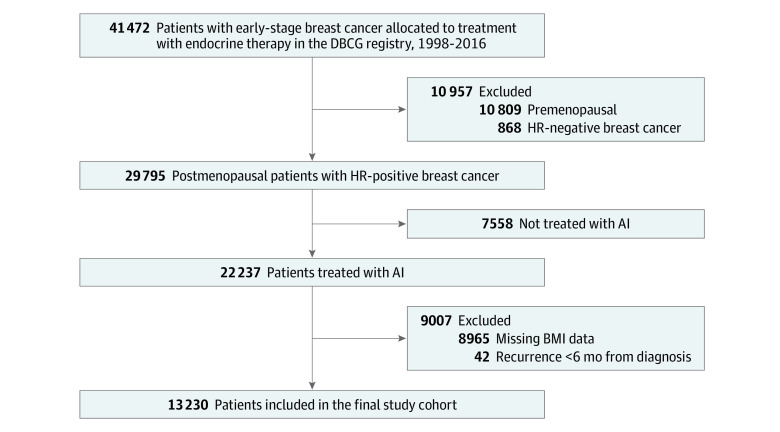
Flowchart of the Study Cohort AIs, aromatase inhibitors; BMI, body mass index; DBCG indicates Danish Breast Cancer Group; HR, hormone receptor.

We identified all postmenopausal women with a primary diagnosis of stage I to III HR+ breast cancer who were reported to the DBCG clinical database from January 1, 1998, until December 31, 2016 (Figure 1). Patients were also required to be assigned to endocrine therapy with AIs in the DBCG database and have information on BMI to be included in the study population.

### Overweight and Obesity Definition

The World Health Organization (WHO) divides BMI into the following body composition groups: patients with a BMI less than 18.5 are classified as having underweight; BMI between 18.5 and 24.9, healthy weight; BMI between 25.0 and 29.9, overweight; BMI between 30.0 and 34.9, obese; and BMI of 35.0 or greater, severe obesity. Healthy weight served as the reference category for calculation of associations. BMI was recorded at surgery or at first adjuvant treatment administration following the surgery. In Denmark the national treatment guidelines recommend the initiation of adjuvant treatment within 13 days of breast cancer surgery.^29^

### Definition of Analytic Variables

Age at breast cancer diagnosis was categorized in decades for descriptive purposes and included as a continuous variable in multivariable models. Histological grade was modeled and described as a categorical variable (grades I, II, and III and not graded). We defined 3 categories of tumor size in millimeters for descriptive and analytical purposes: less than 10 mm, 10 to 20 mm, and more than 20 mm. CCI was categorized as no comorbidity (CCI, 0), moderate comorbidity (CCI, 1-2), and severe comorbidity (CCI, >2); this was described and modeled as a categorical variable. Dichotomous variables included lymph node status (positive [present metastatic lymph nodes] or negative [no metastatic lymph nodes]), *ERBB2* status (negative or positive), and cancer treatments, ie, surgery type (mastectomy or breast conserving), adjuvant radiotherapy (yes or no), and adjuvant chemotherapy (yes or no).

### Outcome

The end point was breast cancer recurrence, defined as the time from 6 months after the date of breast cancer surgery until the earliest occurrence of any breast cancer recurrence recorded. Data on breast cancer recurrences were retrieved from the DBCG.^21^ Clinical follow-up of patients in DBCG ends after 10 years of follow-up. We used a validated algorithm to identify breast cancer recurrences that the DBCG registry had missed (eAppendix 2 in Supplement 1).^30^

### Statistical Analysis

Follow-up for breast cancer recurrence began 6 months after breast cancer surgery and continued until the first event of breast cancer recurrence, contralateral breast cancer, new primary malignant neoplasm, death, emigration, end of clinical follow-up at 10 years, or September 25, 2018. Patients with these events were censored when the event occurred. The median follow-up was calculated using the reverse Kaplan-Meier approach.^31^

We fit Cox regression models to compute crude and multivariable-adjusted hazard ratios with 95% CIs for breast cancer recurrence according to BMI categories. Only patients with complete data on all regressed variables were included in the analyses. Multivariable models included the following covariates: age at diagnosis, tumor size, CCI, node status, histological grade, surgical procedure, adjuvant chemotherapy, and radiotherapy. Schoenfeld residuals were calculated to test the proportional hazards assumption, which was not violated. In further analyses, we tried to account for the competing risk of contralateral breast cancer, new primary malignancies, and death using the Fine and Gray competing risk regression model to calculate the subdistribution hazard ratio with 95% CIs.^32^

To illustrate the association between BMI and breast cancer recurrence, we first fit a multivariable Cox regression model with BMI as a continuous exposure with 95% CIs. Second, the visualization of the association between continuous BMI and hazard ratios for breast cancer recurrence was performed with restricted cubic splines relative to the cohort median BMI of 25.4. The knots were specified as recommended by Harrell^33^ and placed at BMI 19.5, 23.9, 27.4, and 36.1 (5th, 35th, 65th, and 95th percentiles).^34^

Analyses were performed using Stata version 17 (StataCorp) between November 2022 and April 2023. No hypothesis testing was executed in this observational study.

## Results

We enrolled a cohort of 13 230 postmenopausal patients with primary, early-stage, HR+ breast cancer. Median (IQR) age at diagnosis was 64.4 (58.6-70.2) years. Median (IQR) recurrence follow-up was 6.2 (3.6-8.5) years, and the total person-years of recurrence follow-up was 73 904, over which 1587 recurrences occurred.

At diagnosis, 296 patients (2.2%) had underweight, 5873 patients (44.4%) had healthy weight, 4294 (32.5%) had overweight, 1909 (14.4%) had obesity, and 858 (6.5%) had severe obesity. Patients with healthy weight and overweight were older than patients with obesity and severe obesity at diagnosis. Most patients had a low CCI score, but patients with overweight, obesity, and severe obesity had more comorbidities at diagnosis than patients with healthy weight. Patients with overweight, obesity, and severe obesity had tumors with a higher histological grade, were more likely to have lymph node involvement at diagnosis, and had larger tumors compared with patients with healthy weight. Patients who had overweight, obesity, and severe obesity were also more likely to undergo breast-conserving surgery and be treated with adjuvant chemotherapy and/or radiotherapy (Table 1).

**Table 1.** Characteristics of 13 230 Danish Patients With Breast Cancer According to BMI

Characteristics	Patients, No. (%)^a^					
	Total (N = 13 230)	Underweight (BMI <18.5) (n = 296)	Healthy weight (BMI 18.5-24.9) (n = 5873)	Overweight (BMI 25.0-29.9) (n = 4294)	Obesity (BMI 30.0-34.9) (n = 1909)	Severe obesity (BMI ≥35.0) (n = 858)
Year of surgery						
1999	<15 (NA)	NA	<5 (NA)	6 (0.1)	<5 (NA)	<5 (NA)
2000	<25 (NA)	<5 (NA)	<15 (NA)	11 (0.3)	<5 (NA)	<5 (NA)
2001	<45 (NA)	<5 (NA)	<20 (NA)	11 (0.3)	<10 (NA)	<10 (NA)
2002	<120 (NA)	<5 (NA)	<70 (NA)	34 (0.8)	<15 (NA)	<5 (NA)
2003	230 (1.7)	10 (3.4)	141 (2.4)	48 (1.1)	23 (1.2)	8 (0.9)
2004	202 (1.5)	5 (1.7)	80 (1.4)	68 (1.6)	38 (2.0)	11 (1.3)
2005	462 (3.5)	8 (2.7)	225 (3.8)	134 (3.1)	58 (3.0)	37 (4.3)
2006	526 (4.0)	14 (4.7)	248 (4.2)	156 (3.6)	77 (4.0)	31 (3.6)
2007	886 (6.7)	22 (7.4)	408 (6.9)	305 (7.1)	103 (5.4)	48 (5.6)
2008	1048 (7.9)	29 (9.8)	454 (7.7)	340 (7.9)	159 (8.3)	66 (7.7)
2009	1567 (11.8)	40 (13.5)	677 (11.5)	515 (12.0)	226 (11.8)	109 (12.7)
2010	1498 (11.3)	26 (8.8)	638 (10.9)	496 (11.6)	246 (12.9)	92 (10.7)
2011	1416 (10.7)	31 (10.5)	606 (10.3)	485 (11.3)	201 (10.5)	93 (10.8)
2012	1082 (8.2)	20 (6.8)	490 (8.3)	370 (8.6)	149 (7.8)	53 (6.2)
2013	1281 (9.7)	32 (10.8)	575 (9.8)	404 (9.4)	179 (9.4)	91 (10.6)
2014	1430 (10.8)	23 (7.8)	645 (11.0)	457 (10.6)	197 (10.3)	108 (12.6)
2015	827 (6.3)	16 (5.4)	336 (5.7)	274 (6.4)	146 (7.6)	55 (6.4)
2016	580 (4.4)	15 (5.1)	251 (4.3)	180 (4.2)	89 (4.7)	45 (5.2)
Age at diagnosis, y						
<50	105 (0.8)	<5 (NA)	58 (1.0)	19 (0.4)	15 (0.8)	10 (1.2)
50-59	4060 (30.7)	88 (29.7)	1836 (31.3)	1285 (29.9)	570 (29.9)	281 (32.8)
60-69	5659 (42.8)	108 (36.5)	2458 (41.9)	1820 (42.4)	869 (45.5)	404 (47.1)
70-79	2642 (20.0)	71 (24.0)	1151 (19.6)	901 (21.0)	377 (19.7)	142 (16.6)
>80	764 (5.8)	<30 (NA)	370 (6.3)	269 (6.3)	78 (4.1)	21 (2.4)
Charlson Comorbidity Index score						
0	9603 (72.6)	186 (62.8)	4465 (76.0)	3112 (72.5)	1312 (68.7)	528 (61.5)
1-2	2942 (22.2)	87 (29.4)	1133 (19.3)	979 (22.8)	478 (25.0)	265 (30.9)
≥3	685 (5.2)	23 (7.8)	275 (4.7)	203 (4.7)	119 (6.2)	65 (7.6)
Histological type						
Ductal	10 952 (82.8)	245 (82.8)	4861 (82.8)	3563 (83.0)	1570 (82.2)	713 (83.1)
Lobular	1588 (12.0)	42 (14.2)	750 (12.8)	474 (11.0)	217 (11.4)	105 (12.2)
Other/missing	690 (5.2)	9 (3.0)	262 (4.5)	257 (6.0)	122 (6.4)	40 (4.7)
Histological grade^b^						
Grade I	2997 (22.7)	69 (23.3)	1442 (24.6)	935 (21.8)	380 (19.9)	171 (19.9)
Grade II	7216 (54.5)	179 (60.5)	3196 (54.4)	2312 (53.8)	1050 (55.0)	479 (55.8)
Grade III	2220 (16.8)	39 (13.2)	920 (15.7)	753 (17.5)	345 (18.1)	163 (19.0)
Not graded or unknown	797 (6.0)	9 (3.0)	315 (5.4)	294 (6.8)	134 (7.0)	45 (5.2)
Lymph node status						
Negative	6277 (47.4)	150 (50.7)	2860 (48.7)	1995 (46.5)	902 (47.2)	370 (43.1)
Positive	6747 (51.0)	<150 (NA)	2931 (49.9)	2227 (51.9)	973 (51.0)	473 (55.1)
Unknown	206 (1.6)	<5 (NA)	82 (1.4)	72 (1.7)	34 (1.8)	15 (1.7)
Tumor size, mm						
<10	1301 (9.8)	<40 (NA)	599 (10.2)	409 (9.5)	174 (9.1)	88 (10.3)
10-20	6610 (50.0)	161 (54.4)	3153 (53.7)	2066 (48.1)	853 (44.7)	377 (43.9)
≥20	4960 (37.5)	100 (33.8)	1974 (33.6)	1695 (39.5)	822 (43.1)	369 (43.0)
Unknown	359 (2.7)	<5 (NA)	147 (2.5)	124 (2.9)	60 (3.1)	24 (2.8)
*ERBB2* status						
Negative	10 032 (75.8)	225 (76.0)	4375 (74.5)	3307 (77.0)	1454 (76.2)	671 (78.2)
Positive	1609 (12.2)	29 (9.8)	743 (12.7)	514 (12.0)	227 (11.9)	96 (11.2)
Missing	1589 (12.0)	42 (14.2)	755 (12.9)	473 (11.0)	228 (11.9)	91 (10.6)
Surgical procedure						
Mastectomy	4449 (33.6)	165 (55.7)	2091 (35.6)	1352 (31.5)	594 (31.1)	247 (28.8)
Breast-conserving surgery	8758 (66.2)	131 (44.3)	3774 (64.3)	2936 (68.4)	1309 (68.6)	608 (70.9)
Allocated to radiotherapy						
No	2534 (19.2)	112 (37.8)	1222 (20.8)	768 (17.9)	317 (16.6)	115 (13.4)
Yes	10 696 (80.8)	184 (62.2)	4651 (79.2)	3526 (82.1)	1592 (83.4)	743 (86.6)
Allocated to chemotherapy						
No	8521 (64.4)	210 (70.9)	3823 (65.1)	2757 (64.2)	1194 (62.5)	537 (62.6)
Yes	4709 (35.6)	86 (29.1)	2050 (34.9)	1537 (35.8)	715 (37.5)	321 (37.4)

Abbreviations: BMI, body mass index (calculated as weight in kilograms divided by height in meters squared); NA, not applicable.

^a^ The masked numbers are not specific as Danish data protection legislation do not allow data to be personally identifiable according to the general data protection regulation.^19^ For the same reason only rounded percentages of distribution is shown for a few variables.

^b^ In total, this cohort includes 32 patients not graded during histological assessment, ie, nonlobular and nonductal breast cancers, which are not histologically graded. These patients are still included in the multivariable models.

**Figure 2.**
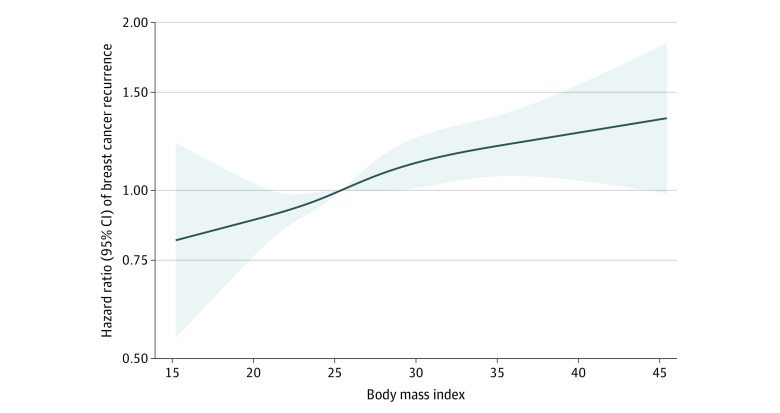
Spline Curve of the Association Between Body Mass Index at Diagnosis and Breast Cancer Recurrence This figure compares hazard ratios for breast cancer recurrence according to body mass index using restricted spline models, adjusting for age at diagnosis, tumor size, Charlson Comorbidity Index, node status, histological grade, surgical procedure, chemotherapy, and radiotherapy. The shaded area represents 95% CIs. The model is relative to the cohort median body mass index of 25.4, with knots specified as recommended by Harrell.^33^ The knots are located at BMI 19.5, 23.9, 27.4, and 36.1 (5th, 35th, 65th, and 95th percentiles).

Patients with underweight had similar breast cancer recurrence hazard to healthy weight patients in multivariable analyses (adjusted hazard ratio, 1.12 [95% CI, 0.77-1.64]). Patients with overweight had a higher risk than those with healthy weight, but the results were not statistically significant (adjusted hazard ratio, 1.10 [95% CI, 0.97-1.24]). There was an association in patients with obesity (adjusted hazard ratio, 1.18 [95% CI, 1.01-1.37]) and severe obesity (adjusted hazard ratio, 1.32 [95% CI, 1.08-1.62]) (Table 2). The association between continuous BMI and breast cancer recurrence estimated via spline regression showed monotonically increasing recurrence hazard at BMIs of 25 or greater (Figure 2).

**Table 2.** Estimates of Recurrence According to BMI at Breast Cancer Diagnosis

Body composition	Patients, No.	Person-years	Recurrences, No.	Crude hazard ratio (95% CI)	Adjusted hazard ratio (95% CI)^a^
Underweight, BMI <18.5	296	1561	28	1.06 (0.73-1.55)	1.12 (0.77-1.64)
Healthy weight, BMI 18.5-24.9	5873	33 117	569	1 [Reference]	1 [Reference]
Overweight, BMI 25.0-29.9	4294	23 995	471	1.16 (1.02-1.31)	1.10 (0.97-1.24)
Obesity, BMI 30.0-34.9	1909	10 518	233	1.31 (1.12-1.52)	1.18 (1.01-1.37)
Severe obesity, BMI ≥35.0	858	4654	110	1.40 (1.14-1.72)	1.32 (1.08-1.62)
Total	13 230	73 904	1587	NA	NA

Abbreviations: BMI, body mass index (calculated as weight in kilograms divided by height in meters squared); NA, not applicable.

^a^ Adjusted for age at diagnosis, tumor size, Charlson Comorbidity Index, node status, histological grade, *ERBB2 *status, surgical procedure, chemotherapy, and radiotherapy.

In analyses considering contralateral breast cancer, new primary malignant neoplasms, and death as competing risks, subdistribution hazard ratios revealed a similar risk of breast cancer recurrence in patients with underweight (subdistribution hazard ratio, 1.14 [95% CI, 0.77-1.67]) as in patients with healthy weight. The risk of breast cancer recurrence was increased in patients with overweight (subdistribution hazard ratio, 1.09 [95% CI, 0.96-1.25]) and obesity (subdistribution hazard ratio, 1.16 [95% CI, 0.99-1.37]), but the results were not statistically significant. There was an association between severe obesity and recurrence (subdistribution hazard ratio, 1.44 [95% CI, 1.17-1.77]) compared with healthy weight (eTable 1 in Supplement 1).

## Discussion

Our study found an association between obesity and increased risks of breast cancer recurrence in postmenopausal patients with HR+ breast cancer treated with AIs in the adjuvant setting. The results of this novel, large population-based cohort study support previous research suggesting that patients with obesity treated with adjuvant AIs may derive less benefit from their adjuvant endocrine therapy than patients with healthy weight.

The biological plausibility of our findings is supported by an abundance of preclinical studies indicating an incomplete suppression of estrogen levels using AIs in HR+ breast cancer when exposed to excess adipose tissue.^35,36,37,38,39,40,41,42,43^ In 2014, a systematic review of observational and interventional studies by Ioannides et al^44^ aimed to assess the association of obesity with AI efficacy in breast cancer treatment. However, no meta-analysis was conducted due to large variability in study factors. The study by Ioannides et al^44^ reported a trend toward a negative association of obesity with AI efficacy, based on data from 8 studies including 13 491 women.^44^ Our current study encompasses a study population of similar size (13 230 participants) and reports an increased risk of recurrence in patients with breast cancer and obesity and allows an estimation of the effect size of obesity on AI efficacy. Ioannides et al^44^ acknowledged the need for more evidence before clinical recommendations are made. Our study contributes additional evidence and encourages further investigations into whether women with obesity and early-stage HR+ postmenopausal breast cancer should be offered endocrine therapies other than the currently recommended AIs to improve their prognosis.

The results of this study are in line with those from the ATAC trial, in which the therapeutic benefit of endocrine treatment decreased with increasing BMI, most evident for patients receiving anastrazole.^4^ Similarly, the BIG 1-98 trial reported poorer survival among breast cancer patients with obesity receiving endocrine therapy but concluded no evidence that the benefit of letrozole—compared with tamoxifen—differed according to BMI.^11^ The differences in the reported efficacy of AIs in the 2 trials according to obesity^4,11^ may be attributed to the different AIs used in the studies (ATAC trial, anastrozole; BIG 1-98, letrozole).^45,46^ In this study, we were not able to differentiate between AIs nor able to compare AIs and tamoxifen.^47^

AIs, like other endocrine therapy regimens, are given to patients based on a standard dosage and one-size-fits-all approach. Smaller studies investigating whether an increased dosage of AIs can improve outcomes among patients with obesity in the metastatic setting^48,49^ showed no additional benefit of an increased dosage of AIs. Yet, these studies were small, conducted in the metastatic setting, and before the implementation of AIs as the first-line adjuvant endocrine therapy for HR+ postmenopausal breast cancer.

Several other factors independent of estrogen may influence breast cancer prognosis among patients with obesity. Our previous research shows shorter disease-free survival among patients with triple-negative breast cancer and overweight.^50^ This suggests that factors such as inflammation,^51,52^ insulin,^53,54^ and dyslipidemia^55,56^ may have a role in the complex interplay between obesity and breast cancer prognosis. In light of this, it is uncertain whether altering endocrine treatment regimens will improve prognosis of patients with obesity. Additional strategies, such as physical activity and diet interventions, may be required.

Following the publication of post hoc analyses of the ATAC trial and the BIG 1-98 trial,^4,11^ this study is the first to revisit the question of whether patients with breast cancer and obesity who are treated with AIs have higher risk of breast cancer recurrence and mortality (eTable 2 and eAppendix 1 in Supplement 1). With obesity on the rise worldwide, our findings give cause for concern and suggest that some patients with breast cancer may derive suboptimal benefit from their cancer treatment. Research attention needs to be directed toward the optimization of care in patients with breast cancer and obesity. To answer whether AIs are inferior to tamoxifen in patients with breast cancer and obesity a study using individual patient data for a meta-analysis of the 2 trials (ATAC and BIG 1-98)^4,11^ could be performed.

There are several ongoing initiatives related to estrogen levels in patients with breast cancer and obesity, ie, studies by Wellberg et al^35,57,58^ on the role of estrogen in preclinical obese breast cancer models and clinical studies by Iyengar et al.^59^ investigating the effect of exercise and plant-based diet on aromatase levels in postmenopausal women with obesity and HR+ breast cancer. Furthermore, the EBBA-II study by Thune et al,^60^ testing a 12-month exercise program in patients with breast cancer, and the BWEL study by Ligibel et al,^61^ investigating weight-loss intervention in patients with breast cancer and obesity, will contribute with information on the impact of exercise on cardiometabolic health and breast cancer outcomes. This study underlines the importance of these projects and encourages new randomized clinical studies to assess whether the choice of endocrine treatment (eg, AIs vs tamoxifen) affects prognosis in patients with breast cancer and obesity.

### Limitations

There are limitations to our study. First, this study includes patients with breast cancer treated with AIs at any point during their adjuvant oncological care. Since data on the date for administration of AIs is not registered, we cannot rule out that some patients intermittently received other adjuvant treatments (eg, tamoxifen). For the same reason, the study lacks data on AI adherence, which may worsen with adiposity. Given this, our study may be prone to misclassification bias. Second, the final study cohort included in the survival analyses only included patients with available data on BMI in the DBCG clinical database or the Danish Anesthesia Database. Although no information on why some patients have missing BMI is available, it needs to be acknowledged that this study population is selected as almost 40% of the postmenopausal patients with HR+ breast cancer treated with AIs had missing data on BMI. Therefore, this study may be prone to selection bias and its results should be carefully interpreted. Patients with missing BMI data were older, had lower histological grade, were less frequently diagnosed with *ERBB2*-positive disease, and were less frequently treated with chemotherapy when compared with patients with BMI data available (eTables 3-5 in Supplement 1). Finally, the years of patient inclusion incorporated a period when AIs were not the established guideline endocrine therapy. AIs were introduced in Danish clinical practice in 2004 and were offered as sequential treatment to patients treated prior to this (ie, patients treated with tamoxifen).^23^ Some of the patients in the study cohort diagnosed prior to the introduction of AIs in Danish clinical practice were participating in the clinical trial, BIG 1-98.^62^ However, we do not expect breast cancer diagnosis, treatment, or follow-up to be different from patients not in clinical trials given the centralized and robust clinical guidelines provided by DBCG.^23^

## Conclusions

In this study, obesity was associated with an increased risk of breast cancer recurrence and mortality in postmenopausal patients with HR+ early-stage breast cancer treated with AIs. To secure equal treatment regardless of body composition, further research ought to examine whether estrogen suppression is sufficient in patients with breast cancer and obesity treated with AIs.

## Notes

Supplement 1. **eTable 1.** Estimates of Recurrence According to Body Mass Index in Competing Risk Analyses **eTable 2.** Estimates of Breast Cancer Mortality According to Body Mass Index in Survival Analyses **eTable 3.** Patient Characteristics According to Patients With Available BMI Data vs Patients With Missing BMI Data **eTable 4.** Patient Characteristics According to BMI of Patients With BMI Data Available in the Danish Anesthesiology Database **eTable 5.** Patient Characteristics According to BMI of Patients With BMI Data Available in the Danish Breast Cancer Group Registry **eAppendix 1.** Description of the Danish Registry of Causes of Death Used to Define Breast Cancer Mortality **eAppendix 2.** Algorithm Used to Identify Breast Cancer Recurrences Not Captured by the Danish Breast Cancer Group Database Using Other Danish Registries

Supplement 2.
Data Sharing Statement
