# Supplementary material for: Obesity and Risk of Recurrence in Patients With Breast Cancer Treated With Aromatase Inhibitors
Source: JAMA Netw Open. 2023 Oct 13;6(10):e2337780. doi: 10.1001/jamanetworkopen.2023.37780 (PMC10576219; doi:10.1001/jamanetworkopen.2023.37780)
Supplement: Supplement 1. — eTable 1. Estimates of Recurrence According to Body Mass Index in Competing Risk Analyses eTable 2. Estimates of Breast Cancer Mortality According to Body Mass Index in Survival Analyses eTable 3. Patient Characteristics According to Patients With Available BMI Data vs Patients With Missing BMI Data eTable 4. Patient Characteristics According to BMI of Patients With BMI Data Available in the Danish Anesthesiology Database eTable 5. Patient Characteristics According to BMI of Patients With BMI Data Available in the Danish Breast Cancer Group Registry eAppendix 1. Description of the Danish Registry of Causes of Death Used to Define Breast Cancer Mortality eAppendix 2. Algorithm Used to Identify Breast Cancer Recurrences Not Captured by the Danish Breast Cancer Group Database Using Other Danish Registries [file jamanetwopen-e2337780-s001.pdf]

Supplemental Online Content

Harborg S, Cronin-Fenton D, Jensen MR, Ahern TP, Ewertz M, Borgquist S. Obesity and risk of recurrence in patients with breast cancer treated with aromatase inhibitors. *JAMA Netw Open.* 2023;6(10):e2337780. doi:10.1001/jamanetworkopen.2023.37780

eTable 1. Estimates of Recurrence According to Body Mass Index in Competing Risk Analyses

eTable 2. Estimates of Breast Cancer Mortality According to Body Mass Index in Survival Analyses

eTable 3. Patient Characteristics According to Patients With Available BMI Data vs Patients With Missing BMI Data

eTable 4. Patient Characteristics According to BMI of Patients With BMI Data Available in the Danish Anesthesiology Database

eTable 5. Patient Characteristics According to BMI of Patients With BMI Data Available in the Danish Breast Cancer Group Registry

eAppendix 1. Description of the Danish Registry of Causes of Death Used to Define Breast Cancer Mortality

eAppendix 2. Algorithm Used to Identify Breast Cancer Recurrences Not Captured by the Danish Breast Cancer Group Database Using Other Danish Registries

This supplemental material has been provided by the authors to give readers additional information about their work.

© 2023 Harborg S et al. *JAMA Network Open.*

eTable 1. Estimates of Recurrence According to Body Mass Index in Competing Risk Analyses

All HR+ postmenopausal breast cancer patients treated with aromatase inhibitors at any point included in the DBCG from 1998-2016.

**Body composition**	**No. Of patients**	**Person-years**	**Recurrence**	**Subdistributional hazard ratio (95% CI)**	**Adjusted subdistributionalhazard ratio (95% CI)**
Underweight	296	1,561	27	1.04 (0.71-1.53)	1.14 (0.77-1.67)
Healthy-weight	5,873	33,117	496	Ref	Ref
Overweight	4,294	23,995	406	1.14 (1.00-1.30)	1.09 (0.96-1.25)
Obesity	1,909	10,518	202	1.29 (1.10-1.52)	1.16 (0.99-1.37)
Severe obesity	858	4,654	102	1.48 (1.20-1.83)	1.44 (1.17-1.77)
**Total**	13,230	73,904	1,233		

**Adjusted for:* age at diagnosis, tumor size, Charlson comorbidity index, node status, histological grade, HER2-status, surgical procedure, chemotherapy, and radiotherapy.

© 2023 Harborg S et al. *JAMA Network Open.*

eTable 2. Estimates of Breast Cancer Mortality According to Body Mass Index in Survival Analyses

All HR+ postmenopausal breast cancer patients treated with aromatase inhibitors at any point included in the DBCG from 1998-2016.

Body composition	No. Of patients	Person-years	Breast cancer deaths	Crude hazard ratio (95% CI)	Adjusted hazard ratio (95% CI)
Underweight	296	1,806	29	1.50 (1.03-2.18)	1.39 (0.96-2.02)
Healthy-weight	5,873	38,149	416	Ref	Ref
Overweight	4,294	27,468	312	1.06 (0.91-1.22)	1.04 (0.90-1.21)
Obese	1,909	12,051	146	1.13 (0.93-1.36)	1.09 (0.91-1.32)
Severe obesity	858	5,356	73	1.27 (0.99-1.63)	1.33 (1.04-1.71)
**Total**	13,230	84,830	976		

**Adjusted for:* age at diagnosis, tumor size, Charlson comorbidity index, node status, histological grade, HER2-status, surgical procedure, chemotherapy, and radiotherapy.

© 2023 Harborg S et al. *JAMA Network Open.*

eTable 3. Patient Characteristics According to Patients With Available BMI Data vs Patients With Missing BMI Data

	**Total**<br><br>N=22,195	**BMI available**<br><br>N=13,230	**Missing BMI**<br><br>N=8,965
**Year of surgery**			
1998	<5	-	<5
1999	<100	<15	<90
2000	<230	<25	200 (2.2%)
2001	322 (1.5%)	43 (0.3%)	279 (3.1%)
2002	530 (2.4%)	118 (0.9%)	412 (4.6%)
2003	834 (3.8%)	230 (1.7%)	604 (6.7%)
2004	873 (3.9%)	202 (1.5%)	671 (7.5%)
2005	933 (4.2%)	462 (3.5%)	471 (5.3%)
2006	992 (4.5%)	526 (4.0%)	466 (5.2%)
2007	1,219 (5.5%)	886 (6.7%)	333 (3.7%)
2008	1,477 (6.7%)	1,048 (7.9%)	429 (4.8%)
2009	2,093 (9.4%)	1,567 (11.8%)	526 (5.9%)
2010	1,964 (8.8%)	1,498 (11.3%)	466 (5.2%)
2011	1,810 (8.2%)	1,416 (10.7%)	394 (4.4%)
2012	1,824 (8.2%)	1,082 (8.2%)	742 (8.3%)
2013	1,913 (8.6%)	1,281 (9.7%)	632 (7.0%)
2014	1,858 (8.4%)	1,430 (10.8%)	428 (4.8%)
2015	1,731 (7.8%)	827 (6.3%)	904 (10.1%)
2016	1,498 (6.7%)	580 (4.4%)	918 (10.2%)
**Age at diagnosis**			
< 50	147 (0.7%)	105 (0.8%)	42 (0.5%)
50-59	5,840 (26.3%)	4,060 (30.7%)	1,780 (19.9%)
60-69	9,898 (44.6%)	5,659 (42.8%)	4,239 (47.3%)
70-79	4,880 (22.0%)	2,642 (20.0%)	2,238 (25.0%)
> 80	1,430 (6.4%)	764 (5.8%)	666 (7.4%)
**Charlson Co-morbidity Index Score**			
None (CCI=0)	15,921 (71.7%)	9,603 (72.6%)	6,318 (70.5%)
Mild (CCI=1-2)	4,989 (22.5%)	2,942 (22.2%)	2,047 (22.8%)
Severe (CCI≥3)	1,285 (5.8%)	685 (5.2%)	600 (6.7%)
**Histological type**			
Ductal	18,245 (82.2%)	10,952 (82.8%)	7,293 (81.3%)
Lobular	2,682 (12.1%)	1,588 (12.0%)	1,094 (12.2%)
Other/missing	1,268 (5.7%)	690 (5.2%)	578 (6.4%)
**Histological Grade**			
Grade I	5,357 (25.9%)	2,997 (24.1%)	2,360 (28.7%)
Grade II	11,941 (57.8%)	7,216 (58.0%)	4,725 (57.5%)
Grade III	3,353 (16.2%)	2,220 (17.9%)	1,133 (13.8%)
Not graded/Unknown	1,544	797	747
**Lymph node status**			
Negative	10,907 (49.8%)	6,277 (48.2%)	4,630 (52.2%)
Postive	10,987 (50.2%)	6,747 (51.8%)	4,240 (47.8%)
Unknown	301	206	95

© 2023 Harborg S et al. *JAMA Network Open.*

**Tumor size, mm**			
< 10	2,140 (9.9%)	1,301 (10.1%)	839 (9.5%)
10-20	11,266 (51.9%)	6,610 (51.4%)	4,656 (52.6%)
≥ 20	8,311 (38.3%)	4,960 (38.5%)	3,351 (37.9%)
Unknown	478	359	119
**HER2 receptor status**			
Negative	15,879 (88.9%)	10,032 (86.2%)	5,847 (94.0%)
Positive	1,984 (11.1%)	1,609 (13.8%)	375 (6.0%)
Missing	4,332	1,589	2,743
**Surgical procedure**			
Mastectomy	7,618 (34.3%)	4,235 (32.0%)	3,383 (37.7%)
Breast-conserving surgery	14,577 (65.7%)	8,995 (68.0%)	5,582 (62.3%)
**Allocated to radiotherapy**			
No	4,643 (20.9%)	2,534 (19.2%)	2,109 (23.5%)
Yes	17,552 (79.1%)	10,696 (80.8%)	6,856 (76.5%)
**Allocated to chemotherapy**			
No	16,806 (75.7%)	8,521 (64.4%)	8,285 (92.4%)
Yes	5,389 (24.3%)	4,709 (35.6%)	680 (7.6%)

© 2023 Harborg S et al. *JAMA Network Open.*

eTable 4. Patient Characteristics According to BMI of Patients With BMI Data Available in the Danish Anesthesiology Database

	Total<br><br>N=13,230	Underweight (BMI <18.5)<br><br>N=296	Healthy-weight (BMI 18.5-24.9)<br><br>N=5,873	Overweight (BMI 25-29.9)<br><br>N=4,294	Obesity (BMI 30-34.9)<br><br>N=1,909	Severe obesity (BMI ≥35)<br><br>N=858
**Year of surgery**						
2004	<15	-	6 (0.2%)	<5	<5	-
2005	<240	7 (3.5%)	121 (3.1%)	<70	<25	15 (2.8%)
2006	358 (4.2%)	12 (5.9%)	182 (4.7%)	89 (3.3%)	53 (4.6%)	22 (4.1%)
2007	563 (6.7%)	18 (8.9%)	263 (6.8%)	189 (7.0%)	63 (5.4%)	30 (5.6%)
2008	730 (8.6%)	23 (11.4%)	324 (8.4%)	229 (8.5%)	111 (9.6%)	43 (8.0%)
2009	1,082 (12.8%)	27 (13.4%)	482 (12.5%)	352 (13.0%)	149 (12.9%)	72 (13.4%)
2010	903 (10.7%)	16 (7.9%)	421 (10.9%)	277 (10.3%)	145 (12.5%)	44 (8.2%)
2011	896 (10.6%)	24 (11.9%)	382 (9.9%)	309 (11.4%)	120 (10.4%)	61 (11.4%)
2012	783 (9.3%)	12 (5.9%)	371 (9.6%)	262 (9.7%)	102 (8.8%)	36 (6.7%)
2013	1,034 (12.2%)	27 (13.4%)	467 (12.1%)	321 (11.9%)	141 (12.2%)	78 (14.5%)
2014	1,223 (14.4%)	21 (10.4%)	558 (14.4%)	395 (14.6%)	157 (13.5%)	92 (17.1%)
2015	439 (5.2%)	9 (4.5%)	187 (4.8%)	145 (5.4%)	68 (5.9%)	30 (5.6%)
2016	207 (2.4%)	6 (3.0%)	101 (2.6%)	60 (2.2%)	26 (2.2%)	14 (2.6%)
**Age at diagnosis**						
< 50	<60	<5	30 (0.8%)	12 (0.4%)	9 (0.8%)	<5
50 - 59	2,050 (24.2%)	<50	987 (25.5%)	606 (22.4%)	262 (22.6%)	<150
60 - 69	3,750 (44.3%)	73 (36.1%)	1,684 (43.6%)	1,182 (43.8%)	546 (47.1%)	265 (49.3%)
70 - 79	1,965 (23.2%)	56 (27.7%)	851 (22.0%)	681 (25.2%)	276 (23.8%)	101 (18.8%)
> 80	644 (7.6%)	26 (12.9%)	313 (8.1%)	220 (8.1%)	66 (5.7%)	19 (3.5%)
**Charlson Co-morbidity Index Score**						
None (CCI=0)	6,036 (71.3%)	128 (63.4%)	2,906 (75.2%)	1,900 (70.3%)	782 (67.5%)	320 (59.6%)
Mild (CCI=1-2)	2,014 (23.8%)	59 (29.2%)	800 (20.7%)	678 (25.1%)	305 (26.3%)	172 (32.0%)
Severe (CCI≥3)	414 (4.9%)	15 (7.4%)	159 (4.1%)	123 (4.6%)	72 (6.2%)	45 (8.4%)
**Histological type**						
Ductal	7,011 (82.8%)	169 (83.7%)	3,206 (82.9%)	2,232 (82.6%)	963 (83.1%)	441 (82.1%)
Lobular	1,028 (12.1%)	<30	492 (12.7%)	309 (11.4%)	127 (11.0%)	<75
Other/missing	425 (5.0%)	<5	167 (4.3%)	160 (5.9%)	69 (6.0%)	<30
**Histological Grade**						
Grade I	1,962 (24.5%)	24,00 %	980 (26.7%)	589 (23.3%)	234 (21.6%)	112 (22.0%)
Grade II	4,725 (59.1%)	63,00 %	2,138 (58.2%)	1,498 (59.2%)	666 (61.4%)	299 (58.6%)
Grade III	1,311 (16.4%)	14,00 %	557 (15.2%)	444 (17.5%)	184 (17.0%)	99 (19.4%)
Not graded/Unknown	<470	<5	190	170	75	27
**Lymph node status**						

© 2023 Harborg S et al. *JAMA Network Open.*

Negative	4,378 (52.0%)	109 (54.0%)	2,053 (53.4%)	1,368 (50.9%)	590 (51.1%)	48,00 %
Postive	4,041 (48.0%)	93 (46.0%)	1,788 (46.6%)	1,320 (49.1%)	564 (48.9%)	52,00 %
Unknown	<50	-	24	13	5	<5
**Tumor size, mm**						
< 10	869 (10.4%)	18 (8.9%)	422 (11.1%)	257 (9.6%)	115 (10.1%)	57 (10.7%)
okt-20	4,383 (52.5%)	117 (57.9%)	2,130 (55.9%)	1,362 (51.1%)	532 (46.6%)	242 (45.6%)
≥ 20	3,097 (37.1%)	67 (33.2%)	1,256 (33.0%)	1,047 (39.3%)	495 (43.3%)	232 (43.7%)
Unknown	115	-	57	35	17	6
**HER2 receptor status**						
Negative	6,935 (88.9%)	165 (90.7%)	3,117 (88.0%)	2,245 (89.4%)	958 (90.2%)	450 (89.3%)
Positive	866 (11.1%)	17 (9.3%)	426 (12.0%)	265 (10.6%)	104 (9.8%)	54 (10.7%)
Missing	663	20	322	191	97	33
**Surgical procedure**						
Mastectomy	2,557 (30.2%)	110 (54.5%)	1,236 (32.0%)	761 (28.2%)	323 (27.9%)	127 (23.6%)
Breast-conserving surgery	5,907 (69.8%)	92 (45.5%)	2,629 (68.0%)	1,940 (71.8%)	836 (72.1%)	410 (76.4%)
**Allocated to radiotherapy**						
No	1,702 (20.1%)	81 (40.1%)	837 (21.7%)	508 (18.8%)	205 (17.7%)	71 (13.2%)
Yes	6,762 (79.9%)	121 (59.9%)	3,028 (78.3%)	2,193 (81.2%)	954 (82.3%)	466 (86.8%)
**Allocated to chemotherapy**						
No	6,134 (72.5%)	160 (79.2%)	2,791 (72.2%)	1,971 (73.0%)	845 (72.9%)	367 (68.3%)
Yes	2,330 (27.5%)	42 (20.8%)	1,074 (27.8%)	730 (27.0%)	314 (27.1%)	170 (31.7%)

© 2023 Harborg S et al. *JAMA Network Open.*

eTable 5. Patient Characteristics According to BMI of Patients With BMI Data Available in the Danish Breast Cancer Group Registry

	Total<br><br>N=13,230	Underweight (BMI <18.5)<br><br>N=296	Healthy-weight (BMI 18.5-24.9)<br><br>N=5,873	Overweight (BMI 25-29.9)<br><br>N=4,294	Obesity (BMI 30-34.9)<br><br>N=1,909	Severe obesity (BMI ≥35)<br><br>N=858
**Year of surgery**						
1999	<15	-	<5	6 (0.2%)	<5	<5
2000	<25	<5	8 (0.2%)	11 (0.4%)	<5	<5
2001	<45	<5	19 (0.5%)	11 (0.4%)	<10	6 (1.1%)
2002	<120	<5	69 (1.9%)	34 (1.2%)	10 (0.8%)	<5
2003	230 (2.8%)	10 (6.1%)	141 (3.9%)	48 (1.8%)	23 (1.8%)	8 (1.5%)
2004	191 (2.3%)	5 (3.1%)	75 (2.1%)	64 (2.3%)	36 (2.9%)	11 (2.0%)
2005	315 (3.8%)	<5	153 (4.3%)	92 (3.4%)	44 (3.5%)	<25
2006	287 (3.5%)	<5	121 (3.4%)	99 (3.6%)	48 (3.8%)	<20
2007	572 (6.9%)	10 (6.1%)	261 (7.3%)	200 (7.3%)	69 (5.5%)	32 (5.8%)
2008	629 (7.6%)	15 (9.2%)	267 (7.5%)	207 (7.6%)	97 (7.7%)	43 (7.8%)
2009	1,116 (13.5%)	31 (19.0%)	458 (12.8%)	377 (13.8%)	170 (13.6%)	80 (14.6%)
2010	1,174 (14.2%)	21 (12.9%)	484 (13.5%)	399 (14.6%)	190 (15.2%)	80 (14.6%)
2011	936 (11.3%)	14 (8.6%)	389 (10.9%)	337 (12.3%)	141 (11.3%)	55 (10.0%)
2012	508 (6.1%)	9 (5.5%)	214 (6.0%)	182 (6.7%)	74 (5.9%)	29 (5.3%)
2013	519 (6.3%)	12 (7.4%)	242 (6.8%)	151 (5.5%)	76 (6.1%)	38 (6.9%)
2014	641 (7.7%)	9 (5.5%)	281 (7.8%)	207 (7.6%)	94 (7.5%)	50 (9.1%)
2015	527 (6.4%)	7 (4.3%)	216 (6.0%)	171 (6.3%)	99 (7.9%)	34 (6.2%)
2016	439 (5.3%)	11 (6.7%)	180 (5.0%)	138 (5.0%)	73 (5.8%)	37 (6.7%)
**Age at diagnosis**						
< 50	<100	<5	55 (1.5%)	18 (0.7%)	12 (1.0%)	<10
50 - 59	3,480 (42.0%)	73 (44.8%)	1,554 (43.4%)	1,131 (41.4%)	489 (39.1%)	233 (42.4%)
60 - 69	3,252 (39.3%)	60 (36.8%)	1,332 (37.2%)	1,078 (39.4%)	540 (43.1%)	242 (44.1%)
70 - 79	1,196 (14.4%)	22 (13.5%)	519 (14.5%)	415 (15.2%)	180 (14.4%)	60 (10.9%)
> 80	<260	<10	121 (3.4%)	92 (3.4%)	31 (2.5%)	<10
**Charlson Co-morbidity Index Score**						
None (CCI=0)	6,247 (75.5%)	110 (67.5%)	2,827 (78.9%)	2,072 (75.8%)	879 (70.2%)	359 (65.4%)
Mild (CCI=1-2)	1,641 (19.8%)	40 (24.5%)	596 (16.6%)	548 (20.0%)	300 (24.0%)	157 (28.6%)
Severe (CCI≥3)	391 (4.7%)	13 (8.0%)	158 (4.4%)	114 (4.2%)	73 (5.8%)	33 (6.0%)
**Histological type**						
Ductal	6,896 (83.3%)	137 (84.0%)	2,981 (83.2%)	2,285 (83.6%)	1,030 (82.3%)	463 (84.3%)
Lobular	957 (11.6%)	20 (12.3%)	436 (12.2%)	292 (10.7%)	145 (11.6%)	64 (11.7%)
Other/missing	426 (5.1%)	6 (3.7%)	164 (4.6%)	157 (5.7%)	77 (6.2%)	22 (4.0%)
**Histological Grade**						
Grade I	1,731 (22.3%)	34 (21.7%)	792 (23.5%)	566 (22.2%)	239 (20.5%)	100 (19.1%)
Grade II	4,427 (57.0%)	95 (60.5%)	1,926 (57.1%)	1,435 (56.4%)	663 (56.8%)	308 (58.9%)

© 2023 Harborg S et al. *JAMA Network Open.*

Grade III	1,607 (20.7%)	28 (17.8%)	653 (19.4%)	545 (21.4%)	266 (22.8%)	115 (22.0%)
Not graded/Unknown	514	6	210	188	84	26
**Lymph node status**						
Negative	3,441 (42.5%)	52,00 %	1,512 (43.0%)	1,125 (42.1%)	519 (42.5%)	201 (37.5%)
Postive	4,662 (57.5%)	48,00 %	2,002 (57.0%)	1,546 (57.9%)	703 (57.5%)	335 (62.5%)
Unknown	<180	<5	67	63	30	13
**Tumor size, mm**						
< 10	745 (9.4%)	11,00 %	331 (9.6%)	244 (9.3%)	104 (8.7%)	48 (9.1%)
okt-20	3,976 (50.0%)	52,00 %	1,852 (53.8%)	1,276 (48.7%)	535 (44.7%)	230 (43.5%)
≥ 20	3,225 (40.6%)	37,00 %	1,260 (36.6%)	1,099 (42.0%)	557 (46.6%)	251 (47.4%)
Unknown	<340	<5	138	115	56	<25
**HER2 receptor status**						
Negative	5,783 (81.2%)	111 (81.6%)	2,437 (80.2%)	1,941 (81.5%)	887 (82.1%)	407 (84.3%)
Positive	1,336 (18.8%)	25 (18.4%)	602 (19.8%)	440 (18.5%)	193 (17.9%)	76 (15.7%)
Missing	1,16	27	542	353	172	66
**Surgical procedure**						
Mastectomy	2,783 (33.6%)	87 (53.4%)	1,293 (36.1%)	859 (31.4%)	388 (31.0%)	156 (28.4%)
Breast-conserving surgery	5,496 (66.4%)	76 (46.6%)	2,288 (63.9%)	1,875 (68.6%)	864 (69.0%)	393 (71.6%)
**Allocated to radiotherapy**						
No	1,436 (17.3%)	61 (37.4%)	689 (19.2%)	436 (15.9%)	188 (15.0%)	62 (11.3%)
Yes	6,843 (82.7%)	102 (62.6%)	2,892 (80.8%)	2,298 (84.1%)	1,064 (85.0%)	487 (88.7%)
**Allocated to chemotherapy**						
No	3,971 (48.0%)	88 (54.0%)	1,705 (47.6%)	1,328 (48.6%)	594 (47.4%)	256 (46.6%)
Yes	4,308 (52.0%)	75 (46.0%)	1,876 (52.4%)	1,406 (51.4%)	658 (52.6%)	293 (53.4%)

© 2023 Harborg S et al. *JAMA Network Open.*

eAppendix 1. Description of the Danish Registry of Causes of Death Used to Define Breast Cancer Mortality

Helweg-Larsen K. The Danish Register of Causes of Death. Scand J Public Health. 2011;39(7_suppl):26-29. doi:10.1177/1403494811399958

The Danish National Board of Health has kept a register of all Danish deaths since 1875; records became individualized and digital in 1970. Since 1994, the Danish Registry of Causes of Death includes ICD-10 codes to classify causes of death according to the World Health Organization's guidelines. The registry records the date of death with underlying and contributing causes registered by the physician responsible for completing the death certificate.

Definition of breast cancer mortality as an endpoint

In this study a patient was considered have an event of breast cancer mortality if the physician had registered breast cancer as one of the three main causes of death. This as the use of breast cancer mortality as an endpoint is limited by the variance in post-mortem registration by individual physicians, potentially incorrectly assessing the primary cause of death. Information regarding breast cancer mortality was retrieved using the Danish Registry of Causes of Death and defined as the time from six months after breast cancer surgery until death from breast cancer.

Description of statistical analyses used to estimate hazard ratios for breast cancer mortality

Follow-up for mortality began six months after breast cancer surgery and continued until the first event of breast cancer mortality, contralateral breast cancer, new primary malignancy, death, emigration, or September 25, 2018. Patients with these events were censored when the event occurred. We used Cox regression models to compute crude and adjusted hazard ratios (HRs) with 95% confidence intervals (95% CI) for breast cancer mortality according to body composition as defined by BMI. Only patients with complete data on all regressed variables were included in the analyses. The model included the following covariates: age at diagnosis,

© 2023 Harborg S et al. *JAMA Network Open.*

Charlson comorbidity index, tumor size, node status, histological grade, surgical procedure, adjuvant chemotherapy, and radiotherapy.

© 2023 Harborg S et al. *JAMA Network Open.*

eAppendix 2. Algorithm Used to Identify Breast Cancer Recurrences Not Captured by the Danish Breast Cancer Group Database Using Other Danish Registries

Algorithm retrieved from Cronin-Fenton, D., Kjærsgaard, A., Nørgaard, M. et al. Breast cancer recurrence, bone metastases, and visceral metastases in women with stage II and III breast cancer in Denmark. Breast Cancer Res Treat ***167****, 517–528 (2018).* https://doi.org/10.1007/s10549-017-4510-3

The following approach was applied to the data:

1. DNRP-registered or DCR-registered metastases code (ICD10: DC76–DC80) 180 or more days after first breast cancer surgery, and without a new primary cancer diagnosis registered in the DNRP or DCR between the date of the first breast cancer surgery and the date of the DNRP or DCR metastases code. Here and below, a new primary cancer was defined as a new cancer that is different from non-melanoma skin cancer (ICD10 C44).
2. Pathology Registry SNOMED combinations recorded 180 or more days after first breast cancer surgery, and without a new primary cancer diagnosis registered in the DNRP or DCR. Combinations were (1) T code (topography/location) in the breast (T04000-T09420) with morphology codes M8 or M9 with ≥3 in the fifth position (*e.g.*, M8XXX3), (2) any T code with morphology codes M8 or M9 with the numbers 4, 6 or 7 in the fifth position.
3. A code specific for local breast cancer recurrence in the DNRP any time after primary diagnosis: DC509X (these codes have only been used in DNRP beginning in 2012). A code for "recurrence operation" (KHAF) in the DNRP any time after diagnosis.

© 2023 Harborg S et al. *JAMA Network Open.*
