# Supplementary material for: Obesity and Risk of Recurrence in Patients With Breast Cancer Treated With Aromatase Inhibitors
Source: JAMA Netw Open. 2023 Oct 13;6(10):e2337780. doi: 10.1001/jamanetworkopen.2023.37780 (PMC10576219; doi:10.1001/jamanetworkopen.2023.37780)
Supplement: Supplement 2. — Data Sharing Statement [file jamanetwopen-e2337780-s002.pdf]

Data Sharing Statement

Harborg. Obesity and Risk of Recurrence in Patients With Breast Cancer Treated With Aromatase Inhibitors. *JAMA Netw Open*. Published October 13, 2023. doi:10.1001/jamanetworkopen.2023.37780

Data
Data available: No

Additional Information
Explanation for why data not available: The data that support the findings of this study are available to academic parties through project applications to the Danish Clinical Quality Program – National Clinical Registries (RKKP) at https://www.rkkp.dk/.
